# Awareness of and subjective needs for post-discharge healthcare services among older adult patients

**DOI:** 10.1186/s12912-023-01247-3

**Published:** 2023-04-18

**Authors:** Hsin-Yen Yen, Doresses Liu, Mei-Ju Chi, Hao-Yun Huang

**Affiliations:** 1 School of Gerontology and Long-Term Care, College of Nursing, Taipei Medical University, 250 Wuxing St, Taipei, 11031 Taiwan; 2 Department of Nursing, Wan Fang Hospital, Taipei Medical University, Taipei, Taiwan; 3 Center for Nursing and Healthcare Research in Clinical Practice Application, Wan Fang Hospital, Taipei Medical University, Taipei, Taiwan; 4 International Ph.D. Program in Gerontology and Long-Term Care, College of Nursing, Taipei Medical University, Taipei, Taiwan; 5 Registered Nurse, Gold Coast University Hospital, Southport, QLD Australia; 6 Research Center in Nursing Clinical Practice, Wan Fang Hospital, Taipei Medical University, Taipei, Taiwan

**Keywords:** Awareness, Demand, Post-discharge healthcare services, Unmet need, Older adult, Discharge planning

## Abstract

**Background:**

Increasing patient awareness of post-discharge care resources is an effective strategy to reduce rehospitalization rates and medical costs. Therefore, the purpose of this study was to explore hospitalized older adult patients’ awareness of and subjective demands for post-discharge healthcare services.

**Methods:**

A cross-sectional study design was conducted from November 2018 to May 2020. STROBE statement was completed. Participants were inpatients over 65 years of age in the general ward of a medical center in northern Taiwan. A questionnaire was used to collect data by face-to-face interviews. Two hundred and twelve participants were recruited. Home nursing care, home rehabilitation, home respiratory therapy, home services, assistive devices rental, and transportation were the main post-discharge healthcare services in this study.

**Results:**

Overall, 83.5% of older adult patients were aware of and 55.7% of the older adult patients demanded at least one post-discharge healthcare services. Logistic regression results found that, patients experiencing moderate to severe disability and cognitive impairment, and those hospitalized in the past year had significantly higher demands for services.

**Conclusions:**

Developing post-discharge healthcare services for older adult patients provides continuous patient-centered services for assisting patients and their families in adapting to the transition period of the post-acute stage. Satisfying these demands is beneficial for older adult patients and their families, as well as for reducing readmissions and medical costs.

## Introduction

Hospitalization is one of the important cause of decreased activities of daily living (ADLs) in elderly populations. Approximately 30%~60% of older adult patients lose part of their ADL function due to acute conditions and these are often addressed by different models of care management during hospitalization, including discharge planning services and follow-up care after discharge [1]. Older adult patients more likely have adverse health events after discharge, such as unplanned readmissions, increased medical costs, and even death [2–4].

## Background

Post-discharge healthcare services (PDHSs) aim to improve the efficiency and quality of medical care units, assist patients in more quickly adapting to the process of transferring from the hospital to home care or another healthcare facility, and provide post-discharge-related information for patients in need. Developing PDHSs from discharge planning services and transitional care management is important for reducing re-hospitalizations in developed countries. PDHSs provide continuous care for patients, reduce the occurrence of unexpected problems, and reduce potential medical burdens [5–7]. A meta-analysis of protective factors for heart failure patients after discharge from adverse events showed that nurses’ home visits were most effective on reducing post-discharge mortality and readmission rates [8].

Previous studies have shown that most patients still have unmet health care demands after discharge. Older adult patients have higher rates of unmet healthcare demands [9, 10]. Medical staff do not have the time and in some cases the experience necessary to evaluate patients’ demands for home-based services after discharge and thus, miss an opportunity to promote recovery [11, 12]. In addition, patients’ functions and demands after discharge from the hospital are often overlooked, especially living arrangements, the home environment, self-care ability, and caregivers’ skills [11]. For example, nursing staff often overestimate a patient’s ability to carry out ADLs, resulting in insufficient patient prognoses or inappropriate arrangements of care services in the post-acute stage [12].

Patients’ demands after discharge are mainly determined by individual patient’s acute and chronic medical problems, physical functions, potential needs for rehabilitation, their ability to make decisions, and other social environmental factors. In order for patients to receive continuing care after discharge from the hospital to home, medical providers have to consider patient factors, including a patient’s cognitive status and ability to carry out ADLs, the safety of a patient’s living place after discharge, the availability of co-resident assistance, the ability to receive care services, accessibility of transportation for home and hospital visits, and the possibility of continuing patient care from resources in the community [13, 14].

Appropriate PDHS arrangements and connections might improve patients’ health outcomes and satisfaction, reduce the risk of rehospitalization and medical utilization, effectively assist patients in transitioning from hospitalization to the community, and enhance their functional recovery [15]. However, high-risk older adult patients often face problems after discharge, such as not knowing where they can get continuing care services, being unable to get sufficient care services, and not knowing how much time they need to recover, what form of rest they need, what activities they can perform, where they can find information they need, how to reduce pain, or how to apply for home care services. Some patients also mentioned that they cannot take care of themselves due to restrictions on activities after discharge. After discharge, 90% of patients suffer from physical disturbances, 60%~70% of patients suffer from emotional distress, and 20%~40% of patients have more than one unmet demand [16, 17]. Compared to adults under 65 years of age, older adult patients are more likely to have multiple chronic diseases or debilitating problems, including weight loss, a poor walking ability, increased risk of falls, and declines in muscle endurance. Elderly populations need longer recovery times and more-active, complete, and continuous PDHSs [18].

In Taiwan, the continuous care system that connects the post-acute stage is not complete. Taiwan’s long-term care service system has been in operation for 15 years, the eligibility criteria were focused on functional limitations and age, and most of the service targets are community-dwelled elders, only a few were from hospitals. Care needs that are more likely to occur in the short period after the elderly patient was discharged include home nursing care, home rehabilitation, home respiratory therapy, home services, assistive device rental, and transportation. These service were included in the seventeen service items of the long-term care system in Taiwan. Applying for long-term care services need to be assessed by the care manager, after confirming the care plan, and then the care manager will assist in the linkage of the service. Users need to apply services by themselves and bear part of the cost. However, discharged patients can only rely on the discharge planning service in the hospital to provide relevant care information, or search and access it by themselves. When patients or their families have insufficient awareness of care information, it is more difficult to find suitable services and tends to result in poor care outcomes. It shows that Taiwan’s long-term care system does not have a sufficient connection for the follow-up care of discharged patients.

## Aims of the study

Understanding the difficulties and problems encountered after discharge from the patient’s point of view would be helpful in developing PDHSs as follow-up care strategies. Planning and arranging follow-up care services before discharge are important for obtaining continuous care. Before discharge, providing a self-care plan for symptom management, planning clearer discharge guidance, and increasing the patient’s awareness of PDHS resources would be beneficial strategies for reducing rehospitalization rates and medical costs of discharged patients [19–22]. Therefore, the following questions were proposed by this study:

① What is the status of hospitalized older adult patients’ or their caregivers’ awareness of PDHSs ?

② What is the status of hospitalized older adult patients’ subjective demands for PDHSs ?

③ What are the factors related to the awareness of and demands for PDHSs, including the demographics of older patients, hospitalization status, health status, ADLs, and the characteristics of post-discharge care?

## Methods

### Study design and sampling

For this study, we conducted a cross-sectional analysis from November 2018 to May 2020. Participants were recruited from general wards of a medical center (a third-level public hospital) in Taiwan, were over 65 years of age, were hospitalized in the general wards, and had no hospitalization records within 3 months before the current hospitalization. Patients with a diagnosis of mental illness or congenital malformations were excluded. After recruitment, names of participants who met the criteria were placed on a list. The sampling process was conducted by randomly selecting three patients from the list with random numbers and the potentially eligible patients were almost ten to twenty every day. After selecting, the nurse entered the ward to explain the purpose of the study and confirmed the willingness of the patients to participate in the study. The sample size calculation was based on the anticipated proportion of knowledge of PHDSs in older patients who were discharged from the hospital. The rate of insufficient awareness with PHDSs (p) was 0.2 [16], the significance level was 0.05, and the margin of error (e) was 0.5. The sample size (*n*) was calculated according to the formula: n = z^2^ * p * (1 - p) / e^2^, and the required number of samples for the analysis was 246 [23]. STROBE statement of the cross-sectional study was completed for this study.

### Data collection and procedures

The day before discharge, our interviewer approached a patient in the ward and explained the survey. Data were collected by one-on-one face-to-face interviews. The interviewees were mainly the patients themselves. If a patient had cognitive impairment, the main caregiver was interviewed as a proxy and answered the questions based on the patient’s status. If the interview questions were answered by the proxy, questions about the awareness of the care were answered based on the knowledge of the proxy, but the demand for care was still answered based on the actual health status of the patient. All study interviews were conducted by a single interviewer who was a nurse of the discharge planning care of the hospital to ensure the consistency of inter-rater reliability.

### Instruments and measures

The content of the questionnaire in this study and the operational definition of the main variables were as follows.

① Demographic characteristics: age, gender, educational level, work status, marital status, and physical disabilities or major injury.
② Hospitalization status at this time: hospitalization department (internal medicine, surgery), common comorbidities, having undergone surgery, urination and defecation status, and tube use.
③ Health status: body-mass index (BMI), cognitive functions, hospitalization in the past year, and risky health behaviors (including drinking and smoking habits). We asked two questions to the respondent “Does the patient smoke/drink (yes/no)” and “how often does the patient smoke/drink (often/occasionally/seldom/never)” to represent risky behaviors. When the respondent answered “smoke/drink often”, we categorized them as “having smoking/drinking habits” and combined having either smoking or drinking habits into one variable named risky health behaviors. In addition, the interviewer checked the BMI, cognitive functions, and admission records from clinical charts after the patients were interviewed. If a patient’s answer differed from the clinical charts, the interviewer confirmed the result with the patient again to ensure its correctness.
④ Activities of daily living (ADLs): The Rankin Disability Scale has five degrees of disability. Grade 1 indicates no significant disability and able to carry out all usual ADLs without assistance; grade 2 indicates slight disability and unable to carry out some previous activities but able to look after one’s own affairs without much assistance; grade 3 indicates moderate disability requiring some help but able to walk without assistance; grade 4 indicates moderately severe disability as being unable to walk without assistance and unable to attend to bodily needs without assistance; and grade 5 indicates severe disability, including being bedridden and/or incontinent, and requiring constant nursing care and attention [24]. The patient was asked to perform the relevant physical activity by the interviewer to determine their physical functions and abilities, except for bedridden patients.
⑤ Characteristics of post-discharge care: Likert scale was used in the questions. An answer to “How confident are you in performing self-care” of ‘totally not’ was recorded as 0 and of ‘totally yes’ was recorded as 3. An answer to “Can you or your family seek for care information by yourself” of ‘totally not’ was recorded as 0 and of ‘totally yes’ was recorded as 3. Patients who answered “totally yes” to both two questions were categorized as “have self-care skills” and it was considered a binary variable for the analysis. The answer option to “Are there people who live together who can assist with care” was ‘yes’ or ‘no’ to represent family/companion support in our study.
⑥ Awareness of and demands for PDHSs. Awareness of services was based on knowing the contents of PDHSs, including home nursing care, home rehabilitation, home respiratory therapy, home services, assistive devices rental, and transportation. The question of awareness was “Do you know about the services?” When the respondents answered that they knew about the services, our interviewer inquired further about the main contents of the services to judge whether the respondent correctly knew about the services. Results of awareness of services were recorded by our interviewer’s judgment with a ‘yes’ or ‘no’ option. Further, if the respondent’s awareness of the services were correct, our interviewer asked “Do you think you will need these services after discharge?”, and the respondent answered ‘yes’ or ‘no’. Last, we calculated the awareness of and demands for all six services and created the variables of “awareness of at least one PDHS” and “demand for at least one PDHS”. These two variables were categorized as ‘yes’ or ‘no’, and we implemented them as binary dependent variables.

### Ethical considerations

In accordance with the Declaration of Helsinki, participants were given an explanation of the purpose of the study, the method of collecting data, the right to drop out at any time, and assurances of the protection of their privacy. Before the survey, patients, family members, or caregivers agreed to participate in the study and signed an written informed consent form in the hospital. If participants had any questions, those questions were directly answered during the interview, and participants were given a dedicated service phone line to follow-up to resolve any doubts. This study was reviewed by the Taipei Medical University - Joint Institutional Review Board (TMU-JIRB) under the number N201706057.

### Statistical analysis

After data collection, SPSS 18.0 software (SPSS, Chicago, IL, USA) was used for all statistical analyses. Descriptive statistics were performed for participants’ characteristics, disease diagnoses, health statuses, and awareness of and demands for PDHSs by univariate analyses, describing the distribution by the frequency, percentage, mean, and standard deviation (SD). Chi-squared tests were performed to analyze associations of participants’ characteristics, disease diagnoses, and health statuses with awareness of and demands for PDHSs. A multivariate binary logistic regression analysis was performed to explore risk factors of older adult patients with insufficient awareness and with demands for PDHS. Participants who reported had awareness with any services in this study were categorized into “with awareness”, and other were categorized into “without awareness”. Participants who reported had demand with any services in this study were categorized into “with demand”, and other were categorized into “without demand”. Confounding variables were selected from significant factors in the bivariate analysis which were included in the logistic model and final model.

## Results

In total, 300 participants were recruited, of which 212 (70.7%) returned home after discharge and participated in this study. Among the participants of this study, forty-one (19.3%) caregivers was interviewed as a proxy. Among the participants’ demographics, 114 (53.8%) were women, and the average age of all participants was 80.5 ± 8.9 years. Regarding educational levels, 31 (14.6%) participants were illiterate, 87 participants (41.0%) had finished elementary school, 49 (23.1%) participants had a high school degree, and 45 (21.2%) participants had a college degree or above. Regarding the work status, 98 (46.2%) participants had retired, 90 (42.5%) participants were homemakers, and 24 (11.3%) participants had a job; 151 (71.2%) participants had a spouse, and 32 (15.1%) participants had physical disabilities or major injuries.

As to the index hospitalization status, the main medical problem involved general medicine (65.6%), and 34.4% participants had had surgery. As to comorbidities, participants with high blood pressure accounted for the largest number at 70.3%, followed by heart disease (37.3%) and diabetes (33.5%). On average, 1.98 chronic comorbidities per patient were calculated. During the index hospitalization, 30.7% of participants had experiences of incontinence or needed assistance excreting, and 22.2% of participants had required use of at least one tube (nasogastric tube, trachea, or urinary catheter). About one-third (34.0%) of participants needed an assistive device or assistance from others to move around; 79.7% of participants had normal cognitive function, and 20.3% had cognitive functional impairment.

Before hospitalization, participants with risky health behaviors accounted for 12.7%. Participants with a normal range for the BMI accounted for 42.0%, 12.3% were underweight, and 45.8% were overweight. Less than half (42.0%) of participants had a moderate to severe disability (RDS = 4 or 5). After discharge, 85.8% of participants had family members or co-residents who could assist with their care, and only 46.7% of participants reported that they had the ability to take care of themselves (Table 1).

**Table 1:** Demographic characteristics and health statuses (*N* = 212)

Variable		*n (%)*
Gender	Male	98 (46.2)
	Female	114 (53.8)
Educational level	Illiterate	31 (14.6)
	Primary school	87 (41.0)
	High School	49 (23.1)
	College or above	45 (21.2)
Work status	Still working	24 (11.3)
	Retired	98 (46.2)
	Domestic work	90 (42.5)
Marital status	Have a partner	151 (71.2)
	No partner	61 (28.8)
Disability/Major illness	Yes	32 (15.1)
Main diagnosis	General medicine	139 (65.6)
	Surgical	73 (34.4)
Disease history	Hypertension	149 (70.3)
	Heart disease	79 (37.3)
	Diabetes mellitus	71 (33.5)
Operation	Yes	54 (25.5)
Toileting	Incontinent/assistive	65 (30.7)
Tube use	Yes	47 (22.2)
Risky health behaviors	Yes	27 (12.7)
BMI (kg/m^2^)	< 18.5	26 (12.3)
	18.5 ~ 24.9	89 (42.0)
	> 24.9	97 (45.8)
Ambulation	Assistive	72 (34.0)
Hospitalization experience	Yes	146 (48.7)
Rankin disability scale (Grade)	Grade 1	30 (14.2)
	Grades 2 or 3	93 (43.9)
	Grades 4 or 5	89 (42.0)
Cognition	Normal	169 (79.7)
Family/companion support	Yes	182 (85.5)
Self-care ability	Yes	99 (46.7)

SD, standard deviation; BMI, body-mass index. The recommended levels are adapted from the global WHO recommendation of 18.5–24.9 as a normal BMI. (https://www.who.int/europe/news-room/fact-sheets/item/a-healthy-lifestyle---who-recommendations )

Regarding awareness of and demands for PDHSs, results revealed that participants’ awareness of transportation services was the highest, at 73.6%, followed by home services (73.1%) and rental services of assistive devices (67.9%). However, less than half of participants were aware of home respiratory therapy and home nursing care. Overall, 83.5% of respondents were aware of at least one PDHS. In addition, participants’ demands for rental services of assistive devices were the highest, at 41.5%, followed by transportation services (30.7%) and home rehabilitation (25.9%). Overall, 55.7% of participants had at least one demand for PDHSs (Table 2).

**Table 2:** Awareness of and demand for post-discharge healthcare (*N* = 212)

	Aware of PDHS		With a demand for PDHS	
Post-discharge healthcare	*n*	%	*n*	%
Transportation	156	73.6	65	30.7
Home service	155	73.1	54	25.5
Assistance with device rental	144	67.9	88	41.5
Home rehabilitation	116	54.7	55	25.9
Home nursing care	86	40.6	19	9.0
Home respiratory therapy	78	36.8	7	3.3
At least one PDHS listed above	177	83.5	118	55.7

PDHS, post-discharge healthcare service

In the binary analysis, there were no significant associations of awareness of PDHSs with any of the independent variables. It was found that compared to men (49.0%), women had significantly higher demands for PDHSs (61.4%). Demands of participants 75–84 years of age (65.3%) and participants 85 years of age and older (64.1%) were significantly higher than those age 65–74 years of age (33.9%). Participants without a spouse (72.1%), who had been hospitalized in the past year (65.3%), who had cognitive impairment (76.7%), who had moderate to severe impairments in ADLs (77.5%), and who needed assistive devices or other assistance (72.2%) had significantly higher demands. Participants without self-care ability (69.0%) had a significantly higher demand than those with self-care ability (40.4%). There was no significant association of educational level, hospitalization department, with a physical or mental disability or major injury, the BMI, urination and defecation issues, the use of tubes during hospitalization, comorbidities, or having people who lived together to assist with care after discharge with demands for PDHSs (Table 3).

**Table 3:** Bivariate analysis of awareness of and demand for post-discharge healthcare services

Post-discharge healthcare		Aware of PDHS (n = 212)			With a demand for PDHS (n = 212)		
Related factors		*n*	%	*p*	*n*	%	*p*
Gender	Male	79	80.6	0.194	48	49.0	0.047
	Female	98	86.0		70	61.4	
Age (years)	65 ~ 74	49	79.0	0.420	21	33.9	0.000
	75 ~ 84	63	87.5		47	65.3	
	≥ 85	65	83.3		50	64.1	
Marital status	Have a partner	124	82.1	0.265	74	49.0	0.002
	No partner	53	86.9		44	72.1	
Hospitalization in the past year	Yes	78	82.1	0.379	62	65.3	0.008
	No	99	84.6		56	47.9	
Patient cognition	Abnormal	39	90.7	0.113	33	76.7	0.001
	Normal	138	81.7		85	50.3	
RDS	Grades 1 ~ 3	101	82.1	0.294	49	39.8	0.000
	Grades 4 or 5	76	85.4		69	77.5	
Self-care ability	Yes	86	86.9	0.146	40	40.4	0.000
	No	91	80.5		78	69.0	
Ambulation	Assistive	60	83.3	0.554	52	72.2	0.000
	Independent	117	83.6		66	47.1	

Educational level, main diagnosis, disability/major illness, body-mass index, toileting status, tube use, operation, comorbidities, and family/companion support had no significant relationship with awareness of or demand for post-discharge healthcare according to a Chi-squared test

RDS, Rankin Disability Scale

Significantly related factors found in the binary analysis were analyzed by logistic regression. Results showed that participants with moderate to severe disability in ADL functions (odds ratio (OR) = 4.57, 95% confidence interval (CI) = 2.00 ~ 10.45), with an abnormal cognitive status (OR = 2.61, 95% CI = 1.08 ~ 6.32), or who had been hospitalized in the past year (OR = 2.14, 95% CI = 1.13 ~ 4.07) had a significantly higher chance to PDHSs demand (Table 4).

**Table 4:** Factors associated with demand for post-discharge healthcare by a logistic regression model (*N* = 212)

		With a demand for PDHS	
Related factor		OR	95% CI
Age		1.02	0.98 ~ 1.06
Gender (ref: male)	Female	1.47	0.73 ~ 2.96
Marriage status (ref: with a partner)	No partner	1.95	0.89 ~ 4.25
Hospitalization (ref: no)	Yes	2.14*	1.13 ~ 4.07
Patient cognition (ref: normal)	Abnormal	2.61*	1.08 ~ 6.32
RDS (ref: grades 1 ~ 3)	Grades 4 or 5	4.57***	2.00 ~ 10.45
Self-care ability (ref: yes)	No	1.85	0.90 ~ 3.78
Ambulation (ref: assistive)	Independent	1.29	0.55 ~ 3.03

* *p* < 0.05, *** *p* < 0.001

Independent variables were chosen for the logistic regression based on those that were significant in the bivariate results. OR, odds ratio; CI, confidence interval; RDS, Rankin Disability Scale

## Discussion

This study focused on hospitalized older adult patients’ awareness of and demands for PDHSs in Taiwan. Most of the participants had awareness of and half of them had demands for PDHSs. Functional ability, cognition, and hospitalization in the past year were significant factors affecting the demand for PDHSs.

Over four-fifths (83.5%) of participants revealed that they were aware of at least one type of PDHS. In addition, 55.7% of participants had at least one demand for PDHSs. A Dutch study conducted a questionnaire survey within 1 week after discharge and found that about 20% of older adult patients did not understand the contents of healthcare services, including home services or home nursing care. Respondents with demands for personal care, home services, and transportation services after discharge accounted for about 40% [16]. A previous study indicated that most older adult patients or their main caregivers understood PDHSs, but also nearly half of older adult patients had demands for PDHSs, which were similar to results of this study. Studies conducted in the United States also found that 85.9%~97% of middle-aged and older adult patients needed assistance with at least one ADL after discharge, and 22.4%~33.0% of patients had unsatisfied demands for PDHSs [9, 17]. Those studies revealed that there were still many demands and problems during the transition from the hospital to returning home, and vulnerable older adult patients had more-complicated problems.

Transportation, home care, assistive rental advice, and home rehabilitation were the services older patients and their caregivers were most aware of. Compared to those services, awareness of home skilled nursing and home respiratory therapy was lower at around 40%. In our study, the highest demand for PHDSs was assistive rental advice (41.5%), followed by transportation (30.7%). Older patients and their caregivers were more familiar with supportive social services, and they might not know that some medical services can be performed at home instead of in a hospital. A recent study revealed that the usage rate of home clinical care was still low among an older population [25]. A review article showed that if patients or their main caregivers had sufficient awareness of discharge planning and follow-up care services, they were able to better coordinate with medical units and effectively improve the quality of care after discharge [26]. Increasing the public’s awareness of home medical services may develop an effective post-discharge healthcare service system and achieve the goal of continuity of care.

In the current study, participants with moderate or severe disabilities of ADLs, who were cognitively impaired, and who had been hospitalized in the past year had significantly higher demands for PDHSs. Due to limited mobility and the inability to care for themselves, patients may have had high demands for PDHSs. Research from the United States and Japan showed that the need for assistance in ADLs was related to the need for visiting nurse services or home care services [27, 28]. Elderly people with cognitive impairment are more likely to need long-term care, meaning that patients and main caregivers require more service resources to support care. The poor health conditions of older adult patients who have been hospitalized in the past year might increase the chances of being hospitalized again; therefore, their demands for PDHSs were higher. Claims-based data from Medicare showed that home-based medical care was needed by older populations with a high comorbidity burden and dementia [25]. A review paper highlighted that older people tended to have unmet formal or informal care needs related to their physical and psychological health [29]. The key findings of those studies emphasized the importance of developing healthcare and support service models based on the needs of older people, especially of older patients.

Previous studies found that patients who had a spouse living in the same residence had nearly 50% fewer opportunities to receive home-based PDHSs than those without a spouse living in the same residence [30]. Among individuals living alone, 94% had received a home visit related to PDHSs, but only 40% of individuals not living alone had received a home visit [31]. Ethnicity and gender showed significant differences in post-acute-care PDHSs. Compared to whites, African-Americans received fewer hours of home-based PDHSs and had lower rates of successfully transferring to a nursing home or a cardiac rehabilitation facility [32, 33]. Women with the same degree of disability as men had only a 25% chance of being successfully transferred to such facilities [34]. High-risk patients and patients with unmet demands at discharge were less likely to be transferred to expected facilities [5, 35].

In our study, participants’ awareness of PDHSs reached 83.5%, but no significant independent variables were found in the results. This may represent the general public’s awareness of the service, and it may have been because our government has developed home- and community-related care services for nearly 15 years. Because of mass communication media, most citizens have a basic understanding of various contents of the home- and community-related care services, especially for older adult patients and their families. Therefore, it would be an efficient strategy for medical institutions to strengthen patients’ awareness of PDHSs during discharge preparations, by providing adequate service information and nursing education, assisting patients in seeking PDHS resources, and providing continuing care services to satisfy patient demands after discharge from the hospital, all of which can potentially reduce rehospitalization rates and medical costs [22, 36, 37].

## Limitations

Limitations of this study were as follows. First, in order to control the interview time with patients or family members, we only collected information on six common services of PDHS items, including home nursing care, home rehabilitation, home respiratory therapy, home services, rental services of assistive devices, and transportation services. Not all services were included in the study, which may have led to an underestimation of the results. It is recommended that future studies include more community- and home-based care services. Second, some older adult patients might not have been able to independently cooperate with the interview due to their cognition. The interviewer sometimes needed family members to accompany the patient or provide answers. Therefore, some portion of the results on awareness of and demands for PDHSs was not from patients, but from family members’ or caregivers’ judgments of patients’ demands. Post-discharge care work of patients is closely related to both patients and family caregivers. Therefore, it is recommended that awareness of and demands for PDHSs be discussed with both patients and caregivers, and decisions be made together, in order to provide the most suitable plan for patient care after discharge. Third, we did not verify the results of subjective demands with healthcare professionals or conduct a follow-up interview to confirm the actual care demands. The subjective demands of patients or their family might not reflect real demands after hospitalization. Therefore, discussion with medical professionals or follow-up of care demands is recommended to explore the actual care demands of discharged older patients in future studies. Last, the findings from this hospital in northern Taiwan may cannot be generalized to similar hospitals. Since the study population is not representative of all the older adult patients, the generalization of these results is limited.

## Conclusions

Planning and developing post-discharge care service systems for older adult patients to assist them and their families to adapt to and connect with the transitional period in the post-acute stage are effective strategies that benefit older adult patients, medical units, and overall medical costs. In the current age of information technology, informing patients of the contents of services is no longer the main issue. It is important to understand how to connect patients with home/community services to satisfy their demands after discharge and returning home. Through clinical care staff’s assessments, observations, and communication with patients, they can help patients choose appropriate care services, develop and discuss their post-discharge care plans, and contact and provide continuing patient-centered services so that older adult patients with poor ADLs and vulnerabilities can return home with peace of mind.

Increasing awareness and satisfying demands for PDHSs are important tasks that health policy experts and medical institutions should take into consideration. The medical system should begin to reform the medical process for the health and care needs of older patients to ensure that the accessibility and quality of continuous care [38].

## Data Availability

The datasets generated and/or analysed during the current study are not publicly available due to the Institutional Review Board (IRB) but are available from the corresponding author on reasonable request.
